# Editorial: Sensory processing sensitivity research: recent advances

**DOI:** 10.3389/fpsyg.2025.1724138

**Published:** 2025-11-11

**Authors:** Bianca P. Acevedo, Francesca Lionetti

**Affiliations:** 1 Department of Psychological & Brain Sciences, University of California, Santa Barbara, Santa Barbara, CA, United States; 2 Department of Brain and Behavioral Sciences, University of Pavia, Pavia, Italy

**Keywords:** sensory processing sensitivity (SPS), environmental sensitivity (ES), personality, emotion regulation, highly sensitive child scale

For this Research Topic of *Frontiers in Psychology, titled* “*Sensory processing sensitivity research: recent advances*,” we sought to create a collection of recent studies examining the biologically based trait of Sensory Processing Sensitivity (SPS), also known as Environmental Sensitivity (ES). This call requested articles spanning the domains of physiological and neural processes, emotion, personality, temperament, cognitive function, social processes, and the measurement of SPS. The resulting collection of research studies advance our understanding of processes and profiles related to SPS, and its measurement, around the globe and across the lifespan.

Some studies in this Research Topic furthered our understanding of the characteristics and phenotypic expression of SPS. For example, a study by Laros-van Gorkom et al. revealed that SPS is associated with greater creativity and empathy, and most notably with the Aesthetic Sensitivity (AES) domain of SPS, adding to knowledge on the positive aspects of SPS. A study by Bürger et al. revealed two distinct profiles of SPS groups (based on the Big Five measure of personality): confident highly sensitive persons (HSPs)—with average neuroticism, and high openness and extraversion; and vulnerable HSPs—with high neuroticism and openness, and low levels of extraversion. Yet another study revealed cognitive styles associated with SPS, such that higher SPS was associated with low externally oriented thinking (EOT) and stronger fantasy oriented thinking (Jakobson et al.). Additionally, a study by Jagiellowicz et al. revealed that SPS was associated with medication sensitivity, even when controlling for negative affectivity and gender. A study conducted in Spain revealed that in adults, higher SPS was associated with more openness to experience and agreeableness, as well as better coping strategies, but lower levels of daily functioning (Chacón et al.). Yet another study explored profiles of SPS and emotion regulation (ER) revealing three classes of HSPs: Low SPS-High ER, Moderate SPS-Moderate ER, and High SPS-Low ER (Liu and Tian). Similarly, a study by Yano and Oishi revealed that differences in mental health outcomes as a function of ES were related to the use of different ER strategies: refocusing and planning were associated with better mental health among those with higher sensitivity (ES), while blaming others was associated with diminished mental health as a function of higher ES. Studies with children in Italy highlight how parenting (e.g., maternal warmth) affects highly sensitive children's ER strategies, such that those with better parenting (higher in maternal warmth) showed better ER, as a function of SPS (Sperati et al.). Importantly, these studies pointed to new directions for research and practical applications, emphasizing the need to integrate the study of the relational environment, emotional outcomes, and regulatory processes to better understand how individuals' sensitivity impacts how they adjust to different contexts.

Measurement studies of SPS included in this Research Topic furthered our understanding of its dimensions and pointed towards fruitful future directions. For example, one study conducted in Spain revealed that there was low agreement between parents' and teachers' assessments of children's SPS using the Highly Sensitive Child Scale (Costa-López et al.). This study suggests that some phenotypic expressions of SPS may be context dependent and that observer biases in perceptions of SPS exist; and it also highlights the importance of developing objective measures to assess children's sensitivity. In another measurement study of SPS, a validation of the Spanish Sensory Processing Sensitivity Questionnaire (S-SPSQ) in a Chilean sample confirmed the six-factor structure of the scale including: aesthetic sensitivity, sensory discomfort, social affective sensitivity, emotional and physiological reactivity, sensory comfort/pleasure, and sensory sensitivity to subtle external stimuli (Salinas-Quintana et al.).

Other studies in this Research Topic examined how SPS moderates individuals' responsivity to different contexts. For example, a mixed method study revealed that SPS was associated with greater flourishing with increasing nature connectedness; as well as more emotional reactivity and feelings of being different, as a function of a chaotic home environment (Carroll et al.). This study suggests that it is important to consider the impact of not only emotional, but also structural aspects of the environment, to more fully understand the factors associated with HSPs' wellbeing. Yet another study showed how SPS shapes response to post-migration circumstances of uncertainty among asylum seekers, such that those with higher ES and intolerance for uncertainty reported more negative affect/cognitions, relative to less sensitive individuals (Moscardino et al.). Moreover, a study examining mental health as a function of SPS during the pandemic indicated that highly sensitive (high SPS) adolescents showed significant increases in negative emotions across 2020–2022 (Dragone et al.). These studies are useful for understanding the impact of individuals' sensitivity in contexts beyond traditional and Western culture contexts, such as during challenging, unexpected, and/or crisis circumstances. Additionally, thematic interviews with high SPS students revealed six themes related to the academic context: self-definitions; academic experience; study approach; physical, emotional and cognitive states during and after exams; peer relationships, and student-teacher relationships (Saglietti et al.). Also, a study conducted in Spain revealed that SPS was related to better maternal adjustment; and that in expatriate contexts, more sensitive mothers with higher social support reported stronger maternal adjustment (Lagarrigue et al.).

Overall, the collection of studies included in this Research Topic highlight the breadth of research on high sensitivity, and contribute to a more complex understanding of SPS' across the globe and the lifespan, as well as pointing to future directions. Also, some of the studies in this Research Topic have shed light on the extent to which different contexts promote (or hinder) wellbeing among those with high sensitivity. However, there is still much to be understood about the mechanisms underlying SPS, and how wellbeing may be positively affected among those with high sensitivity.

