# Correction: Editorial: Sensory processing sensitivity research: recent advances

**DOI:** 10.3389/fpsyg.2025.1749082

**Published:** 2025-12-01

**Authors:** Bianca P. Acevedo, Francesca Lionetti

**Affiliations:** 1 Department of Psychological & Brain Sciences, University of California, Santa Barbara, Santa Barbara, CA, United States; 2 Department of Brain and Behavioral Sciences, University of Pavia, Pavia, Italy

**Keywords:** sensory processing sensitivity (SPS), environmental sensitivity (ES), personality, emotion regulation, highly sensitive child scale

In the published article, there were instances of incorrect text.

1. The text was displayed as:

“This study suggests that some phenotypic expressions of SPS may be context dependent and that observer biases in perceptions of SPS exist, and it highlights the importance of developing objective measures to assess children's sensitivity.”

The correct text is:

“This study suggests that some phenotypic expressions of SPS may be context dependent and that observer biases in perceptions of SPS exist; and it also highlights the importance of developing objective measures to assess children's sensitivity.”

2. The text was displayed as:

“In another measurement study of SPS, a validation of the Spanish Sensory Processing Sensitivity Questionnaire (S-SPSQ) in a Chilean Sample.”

The correct text is:

“In another measurement study of SPS, a validation of the Spanish Sensory Processing Sensitivity Questionnaire (S-SPSQ) in a Chilean sample.”

3. The text was displayed as:

“Also, some of the studies in this Research Topic have shed light on the extent to which different contexts promote (or hider).”

The correct text is:

“Also, some of the studies in this Research Topic have shed light on the extent to which different contexts promote (or hinder).”

The original version of this article has been updated.

